# Evaluation of the efficacy of bendiocarb in indoor residual spraying against pyrethroid resistant malaria vectors in Benin: results of the third campaign

**DOI:** 10.1186/1756-3305-5-163

**Published:** 2012-08-08

**Authors:** Razaki Ossè, Rock Aikpon, Gil Germain Padonou, Olivier Oussou, Anges Yadouléton, Martin Akogbéto

**Affiliations:** 1 Centre de Recherche Entomologique de Cotonou (CREC), 06 BP 2604, Cotonou, Bénin; 2 Faculté des Sciences et Techniques, Université d’Abomey Calavi, Calavi, Bénin

**Keywords:** *An.gambiae*, EIR, Longevity, IRS, Bendiocarb, Resistance, Benin

## Abstract

**Background:**

Since 2008, the National Malaria Control Program (NMCP) has been engaged in the implementation of indoor residual spraying (IRS) in Benin. The first and second round was a success with a drastic decrease of malaria transmission in areas under IRS. We present here the results of the third round. The purpose of this study was to compare the results of the third round of IRS to those achieved during the first two rounds. A second success of IRS will enable the Government of Benin to extend the strategy to other areas.

**Methods:**

Mosquito collections were carried out in the department of Ouémé where the homes of four districts were treated with bendiocarb. In these districts, more than 350 000 inhabitants were protected by IRS. A fifth untreated district served as control. In the five districts, mosquito collections were organized to follow the dynamics of malaria transmission and possible changes in the behavior of mosquitoes.

**Results:**

A significant reduction in human biting rate was recorded after the third round of IRS, specifically in Adjohoun (89.78%), Dangbo (56.8%) and Missérété (93.22%) where an inhabitant received less than 2 bites of *An. gambiae* per night. During this same time, the entomological inoculation rate (EIR) declined dramatically in all areas under intervention (74.26% reduction). We also noted a significant reduction in longevity, the blood feeding rate of the vectors and an increase in exophily induced by bendiocarb on *An. gambiae* and *Mansonia spp*.

**Conclusion:**

The present study showed, once again, the effectiveness of bendiocarb on anopheles populations resistant to pyrethroids. This product can be recommended in combination with other insecticides for the management of vector resistance to insecticides.

## Background

Despite much effort, malaria remains a serious public health problem in Africa. The estimated number of malaria cases worldwide in 2010 was 216 million and the vast majority (81%) was found in Africa. Africa accounts for 91% of 655,000 malaria deaths registered in 2010 in the world [1]. In Benin, in 2007 malaria accounted for 43% of the cases and was also the primary cause of health facility admissions [2]. However, the international community is not discouraged. The “Roll Back Malaria” initiative launched on July 23rd, 1998 by The WHO and its partners, received a favorable response from heads of state in the North and peer endemic countries of Africa. In addition, malaria is one of the Millennium Development Goals (MDG) as one of the global targets for 2015 and the period 2001–2010 has been declared by the United Nations as«Decade to Roll Back Malaria in developing countries, particularly in Africa».

It is in this context that the National Malaria Control Programme, which fully complies with all of these decisions to implement its strategies against malaria with two major directions in the field of vector control: the universal access to Long Lasting Insecticidal Nets (LLINs) and Indoor Residual Spraying (IRS). But the main problem of the use of impregnated materials is the development of resistance. In recent years, insecticide resistance was widespread in West Africa [3-6], in East Africa [7], in Central Africa [8] and South Africa [9,10].

In addition, N'Guessan *et al.*[11] reported a decrease in the effectiveness of treated nets and indoor residual spraying of lambda-cyhalothrin in experimental huts in areas of high resistance of mosquitoes in southern Benin. If these reports seem worrying, they need to be placed in context as was done in the discussion of this article. This study was carried out at the experimental hut level and it is still difficult to extrapolate what will happen at the community level. Therefore, this paper aims to investigate the effectiveness of indoor residual spraying using bendiocarb at the community level, but in the presence of high resistance of *An. gambiae* to pyrethroids.

Moreover, according to Rowland [12], the implementation of a resistant management strategy should take into account the resistance mechanisms involved. According to this author, the resistance mechanism in the M form of *An. gambiae* is probably the cause of the reduction in the effectiveness of pyrethroid-impregnated materials. In addition, recent results in phase III in Tori-Bossito, Benin, where indoor residual spraying of insecticide has been implemented on a population of ten thousand inhabitants, failed to draw valid conclusions [13]. According to Akogbéto *et al.*[14], the impact of IRS on malaria transmission in Tori-Bossito was not significantly different from the control area (area where children under five years old and pregnant women were protected by LLINs). According to these authors, this insignificant impact is due to the size of populations covered by IRS. A population of ten thousand persons corresponds to a small village. It is therefore essential to study the effectiveness of malaria vector control methods (IRS and LLINs) taking into account two factors: the presence of vector resistance to insecticides and the size of the human population to be protected.

Taking into account the first factor, Zaim *et al.*[15] recommend the search for alternatives to resistance management. Therefore, since 2008 in Benin, the NMCP decided to use non-pyrethroid insecticides in indoor residual spraying in areas of vector resistance to insecticides [16]. For this reason, during the IRS operation launched by NMCP with support from PMI in 2008 in the Ouémé department, bendiocarb was used. The choice of bendiocarb was made after an evaluation in phase II of several insecticides [16].

The second factor taken into account in this study is the size of the population covered. The IRS campaign of Ouémé is a major operation which covered about 500 000 inhabitants.

From July 2008 to April 2010, three rounds of IRS have been implemented. The treatment of the houses was made by a Non-Governmental Organization, the Research Triangle Institute (RTI) with community participation. According to Akogbéto *et al.*[14], the first two rounds were a great success reducing malaria transmission by 94%.

This survey presents the results of the third round of IRS using bendiocarb in Benin. Thus, we followed the dynamics of malaria transmission in area under the third round of IRS. The recorded data were compared with those of the test year. The entomological monitoring focused on the feeding behavior, the physiological age and entomological inoculation rate of anopheline mosquitoes and the feeding behavior of culicine mosquitoes was considered as well.

## Methods

### Study areas

**Figure 1:**
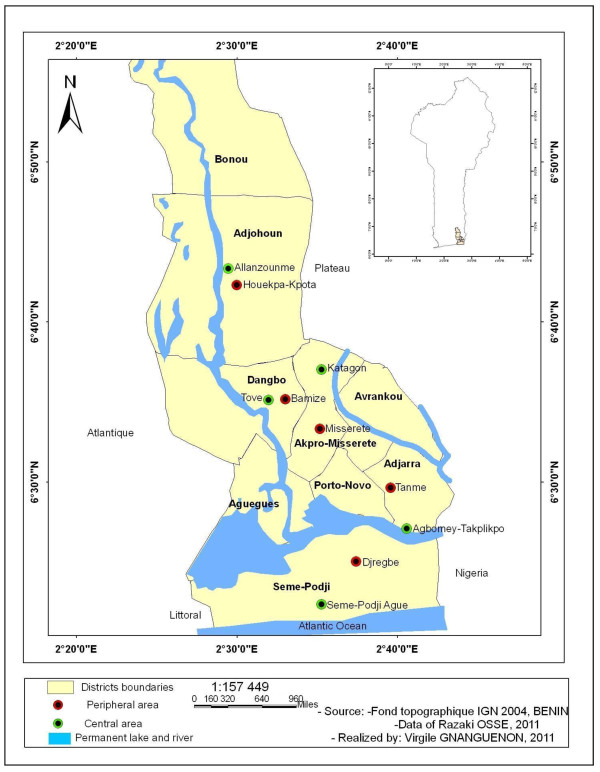
Map of the study area.

The survey was conducted in four districts of Ouémé department (Adjohoun, Dangbo, Missérété and Sèmè) in southern Benin (Figure 1). In each district, two localities were chosen: one located in a central plateau and the other located in the peripheral valley characterized by the presence of temporary and permanent mosquito breeding habitats, respectively. This district has essentially a sub-equatorial climate, with two dry seasons (August-September and December-March) and two rainy seasons (April-July and October-November). The annual mean rainfall is 1,500 mm in July, relative humidity (RH) of 70% ± 5 and the average monthly temperature varies between 23 and 32°C.

The data from these four districts under IRS intervention were compared with those of a control area (district of Adjara) with the same characteristics but where non of the houses had been sprayed. The 4 intervention districts and the control district are located in the same department, the department of Ouémé. The five areas are populated by the same people, the Ouémènou and Goun, who are mostly traders, artisans and officials. The entire population of each district has access to a hospital. Through the efforts of the National Malaria Control Program, access to the targeted coverage with ITNs has become a reality. However, Adjohoun, Dangbo and Sèmè districts are located on the banks of perennial streams. Furthermore, the choice of catch localities took into account the distribution of the breeding sites, except that any disparity in the distribution of breeding sites especially in the districts of intervention and control district could affect the results concerning the impact on mosquito densities. Then, the position of houses relative to permanent and semi permanent breeding sites would be a major criterion of the choice of catch localities in the 5 districts.

In the current promotion of ITNs in all countries of the south Sahara, it is difficult to compare an IRS area, a non-IRS area and non-LLINs area. The four districts under IRS (Adjohoun, Dangbo, Missérété and Sèmè) and the control district (Adjara) belong to the same department (Ouémé). All of the five districts are covered by LLINs in the targeted coverage: Children under 5 years and pregnant women receive LLINs from the NMCP. All the five districts have in common the use of LLINs by both vulnerable populations. The only difference in terms of control interventions is the IRS in Adjohoun, Dangbo, Missérété and Sèmè. Hence, we consider that any reduction in malaria transmission in these four districts in relation to the control district is due to the IRS.

### Implementation of intervention

Before IRS intervention, the choice of an insecticide was necessary. Various insecticides were evaluated over a period of 4 months in experimental huts. Three of them (Sumithion 40 WP [Fenitrothion]; Master Quick ZC [mixture of chlorpyriphos 250 g/l + deltamethrin 12 g/l] and Ficam M [bendiocarb, 800 g/kg]) were effective against mosquitoes resistant to pyrethroids. Of the three insecticides, bendiocarb was the product chosen by the NMCP for the IRS implementation [16].

Indoor residual spraying using bendiocarb was applied at a rate of 400 mg/m² in Adjohoun, Dangbo, Missérété and Sèmè district. The formulation used was the Ficam^TM^ WP in the water-soluble form packaged in sachets of 250 g. Over 90% of households were treated. The first and second IRS operations were made in July 2008 and March 2009 respectively. In 2010, the third round took place in March-April. The three IRS operations were carried out by volunteers recruited from the local community and trained by the Research Triangle Institute (RTI) team, USAID’s implementing partner. The treatment of the walls was done with a manual pressure sprayer of the HUDSON XPERT type. Before treatment, sprayers were protected with a laboratory coat, gloves, boots and a helmet for safety.

### Mosquito sampling

#### Human landing catch

In each district, a central and a peripheral area were selected. Monthly, mosquito collections were carried out from 9 p.m. to 5 a.m. inside and outside houses using a mouth aspirator, by human volunteers who had previously given consent. Two nights of mosquito collections a month were carried out for eight months (before and after intervention). A total of eight night catches were conducted in each locality for a total of 64 night catches per month in the four districts. These catches were made from April to July 2008 (before intervention) and from April to July 2010 (after the third round of IRS). In each district, two houses and four collectors were selected per area for the collection of mosquitoes. The recorded data were used to assess the aggressiveness (HBR), the physiological age and the entomological inoculation rate vectors (EIR).

#### Exit window trap catch

Moreover, in order to assess the impact of interventions on exit induced by the presence of bendiocarb, we sampled mosquitoes using windows exit traps and morning pyrethrum spray catches (PSC). Therefore, in each area, eight bedrooms were selected for mosquito collection in the morning. Exit traps were put over the windows of every bedroom retained. Mosquitoes were collected for 2 nights each month from 6 p.m. to 6 a.m. next day. The houses where the traps were set were selected based on the number of people who slept in them (01 sleeper per hut). The houses were built of mud and wood with a sheet-metal roof and the eve was tight. The area between the upper walls and the roof is closed. The exit window traps used were made of a terylene netting mounted on a 30 cm cubical metallic frame. The entrance side was drawn into a truncated cone with a 2.5 cm diameter hole at its apex, which was 2.5 cm from the opposite face of the trap. In addition to that, mosquitoes resting in the house were collected by Morning Spray Catch (MSC) from 7 a.m. to 9 a.m. The collections from the window traps were done in the morning using a mouth aspirator. Morning pyrethrum spray catches were done using pyrethrum spray and white canvas spread on the floor to collect knocked down mosquitoes. These two sampling methods led to an accurate estimation of the total density of mosquito species in the treated houses and the proportion of female mosquitoes exiting from the houses. Exit rate is estimated by mosquitoes which have escaped treated walls and have been retained in the exit window traps. Anopheles mosquitoes collected were classified according to the state of their abdomens.

*Mansonia spp*. and *Culex spp*. are vectors of viral diseases and filarial nematodes. In the area, a high proportion of these mosquitoes was recorded. Moreover, *Culex quinquefasciatus* is highly resistant to pyrethroids in the study area. In spite of this resistance, the IRS significantly reduced the density of these species of mosquito or increased exophily. These results indicated a successful prospect concerning the use of bendiocarb. We therefore determined the impact of the IRS on the behavior of those mosquitoes.

### Laboratory processing

After each night catch, mosquitoes were sorted by genus as anopheline and culicine and counted. Anophelines were morphologically identified to species using taxonomic keys of Gillies & De Meillon [17] and Gillies & Coetzie [18]. Ovaries from randomly selected female *An.gambiae s.l.* specimens captured on human landing catches were dissected to determine parity rate, by observing the degree of coiling of ovarian tracheoles [19]. Mosquito infectivity rates were determined from head and thorax of all female anopheline specimens by enzyme-linked immunosorbent assay (ELISA) using monoclonal antibodies against *Plasmodium falciparum* circumsporozoite protein (CSP) as described by Wirtz *et al.*[20]. The carcass of these females (abdomens, wing and legs) were stored in individual tubes with silicagel and preserved at −20°C in the laboratory for identification of species and characterization of molecular forms within the *An.gambiae* complex as previously described [21,22].

### Data analysis

The human biting rate [number of bites/man/night] (ma), the sporozoïte rate (Is) and the entomological inoculation rate (EIR) were determined. The percentages of reduction of biting rate (HBR) and EIR were calculated after the intervention. Parturity rates, exophily and blood feeding rate were evaluated. Comparisons of these rates were made by the Chi-square test.

### Ethical consideration

Ethical approval for this study was granted by the Ethical Committee of the Ministry of Health in Benin. The mosquito collectors gave prior informed consent and they were vaccinated against yellow fever. They were also subjected to regular medical check-ups with preventive treatments of malaria.

## Results

### Species and molecular forms of *Anopheles gambiae*

PCR revealed 100% of mosquitoes tested were *Anopheles gambiae s.s.* M form.

### Variation in *An. gambiae* human biting rates in localities under IRS coverage

The results of our research show a lower human biting rate during the third round of IRS in Adjohoun, Dangbo and Missérété districts where every person living in these districts receives less than two bites of *An. gambiae* per night. Indeed, in these districts, the reduction of human biting rate was 89.78% (0.33 bite/man/night after IRS against 3.23 before intervention), 56.8% (1.89 bites against 4.375) and 93.22% (1 bite against 14.75) respectively in Adjohoun, Dangbo and Missérété. However, in the fourth district under IRS intervention (Sèmè), the reduction observed was low, 12.93 bites of *An. gambiae* per man per night after the third round against 24.88 before the intervention (48.03% of reduction) (Table 1).

**Table 1:** Entomological Inoculation Rate (EIR) and Human Biting Rate (HBR) observed before and after IRS intervention

**Localities**	**Variables**	**Before intervention April-July 2008**	**After intervention April-July 2010**	**% Reduction**
**Adjohoun**	Tnber	207	19	
	Man night	64	56	
	HBR	3,23	0,33	89,78
	S%	5,71	10,52	
	EIR (b/m/n)	0,18	0,034	81,11
	EIR/month	5,4	1,02	
	EIR/period	21,6	4,08	
**Dangbo**	Tnber	280	106	
	Man night	64	56	
	HBR	4,375	1,89	56,8
	S%	4,49	2,83	
	EIR (b/m/n)	0,19	0,053	72,11
	EIR/month	5,7	1,59	
	EIR/period	22,8	6,36	
**Missérété**	Tnber	944	56	
	Man night	64	56	
	HBR	14,75	1	93,22
	S%	1,07	1,78	
	EIR (b/m/n)	0,15	0,018	88,13
	EIR/month	4,5	0,534	
	EIR/period	18	2,136	
**Sèmè**	Tnber	1592	724	
	Man night	64	56	
	HBR	24,88	12,93	48,03
	S%	3,5	1,96	
	EIR (b/m/n)	0,87	0,253	70,92
	EIR/month	26,1	7,59	
	EIR/period	104,4	30,36	
**Adjara Control**	Tnber		1117	
	Man night		56	
	HBR		19,95	
	S%		10,36	
	EIR (b/m/n)		2,067	
	EIR/month		62,01	
	EIR/period		248	

* Tnber * Total number, * HBR * Human Bite Rate, * S% * Sporozoite index, * EIR (b/m/n) * Entomological Inoculating Rate (infected bite/man/night).

In the control area, the human biting rate was high: 20 bites per man per night during the same period (April-July 2010).

### Variation of *Mansonia spp* and *Culex spp* human biting rates in localities under IRS coverage

The main mosquitoes which caused a nuisance in the study area were *Mansonia spp*. and *Culex spp*. They were found to be anthropophilic and exophagic. According to our results, the impact of IRS seems low on these 2 Culicinae compared to *An. gambiae*. The reduction of human biting rate observed in *Mansonia spp* and *Culex spp* was low compared to that of *An.gambiae*. With *Mansonia spp*, the reduction was 43.23%, 50.36%, 34.43% and 35% respectively in Adjohoun, Dangbo, Missérété and Sèmè (Table 2). Concerning *Culex spp*, there is no impact. The reduction is too low in the districts of Adjohoun (4.65%), Dangbo (1.36%) and Missérété (8.95%). However, in Sèmè district, it is relatively high: 32% (Table 2).

**Table 2:** **Human Biting Rate (HBR) of* Mansonia spp. *and* Culex spp. *observed before and after IRS intervention**

**Localities**	**Variables**	*** Mansonia spp. ***			***Culex spp***		
		**Before intervention April-July 2008**	**After intervention April-July 2010**	**% Reduction**	**Before intervention April-July 2008**	**After intervention April-July 2010**	**% Reduction**
**Adjohoun**	Tnber	2160	1073		1539	1284	
	Man night	64	56		64	56	
	HBR	33,75	19,16	43,23	24,05	22,93	4,651
**Dangbo**	Tnber	2984	1296		2790	2408	
	Man night	64	56		64	56	
	HBR	46,63	23,14	50,36	43,59	43	1,362
**Missérété**	Tnber	936	537		236	188	
	Man night	64	56		64	56	
	HBR	14,63	9,589	34,43	3,688	3,357	8,959
**Sèmè**	Tnber	2750	1564		1054	630	
	Man night	64	56		64	56	
	HBR	42,97	27,93	35	16,47	11,25	31,69
**Adjara Control**	Tnber		397			433	
	Man night		56			56	
	HBR		7,089			7,732	

### Variation in entomological inoculation rate (EIR) in localities under IRS coverage

Before intervention (April-July 2008), each person received an average of 21.6, 22.9, 18 and 104.4 infected bites over a period of four months, respectively in Adjohoun, Dangbo, Missérété and Sèmè. After the third round of IRS, the rate dropped to 4.08 in Adjohoun, to 6.36 in Dangbo, to 2.136 in Missérété and to 30.36 in Sèmè (Table 1).

The comparison of EIR observed before and after intervention in each district reveals a decrease of 81.1%, 72.11%, 88.13% and 70.92% respectively in Adjohoun, Dangbo, Missérété and Sèmè (Table 1). In the control area, the rate was very high (248 infected bites/man/4 months) (Table 1).

### Variation in the longevity of *An. gambiae* in localities under IRS coverage

For both periods, 1083 ovaries of *An. gambiae* were dissected. Before intervention (April-July 2008), the parity rate of *An. gambiae* was 68.54% in Missérété, 79.94% in Adjohoun. But after the third round of IRS, we observed a dramatic decline in this rate in all areas under intervention (p <0.05). It went from 79.94% to 30% in Adjohoun and 68.34% to 32.14% in Missérété (Table 3).

**Table 3:** **Variation of parity rate of* Anopheles gambiae s.l. * collected during the long rainy season in 2008 before IRS intervention and in the same period after**

**Localities**	**Before intervention (April-July 2008)**		**After intervention (April-July 2010)**		**p-value**
	**Nber diss**	**P rate%**	**Nber diss**	**P rate%**	
**Adjohoun**	70	79,94	10	30	0,001
**Dangbo**	89	78,03	41	46,34	0,000
**Missérété**	78	68,54	28	32,14	0,000
**Sèmè**	114	78,42	244	40,16	0,000
**Control**	93	70,97	316	81,01	0,019
**Total**	444	75,28	639	60,25	0,000

* Nber diss * Number collected and dissected, * IRS * Indoor Residual Spraying, * P rate% * Parity rate%.

### Variation in induced exophily in *An. gambiae* in localities under IRS coverage

Before intervention, rate of induced exophily for Adjohoun, Dangbo, Misserete, and Sèmè was 30.95%, 36.90%, 27.08% and 35.57%, respectively (Table 4). After intervention, the rate of exophily was very high: 81.82% in Dangbo, 80% in Missérété and 75.68% in Sèmè. Compared to natural exophily, the difference was significant in all districts treated (p <0.05). In Adjohoun, no *An.gambiae* were collected in windows traps or in the morning pyrethrum spray catches after intervention.

**Table 4:** **Induced exophily in* An. gambiae *pre and post IRS operation using bendiocarb in 4 study localities**

**Localities**	**Before intervention (May-July 2008)**			**After intervention (May-July 2010)**			**p-value**
	**Total No**	**Nber exit windowtrap**	**Exit rate (%)**	**Total No**	**Nber exit windowtrap**	**Exit rate (%)**	
**Adjohoun**	84	26	30,95	0	0	-	-
**Dangbo**	84	31	36,9	0	18	81,82	< 0,05
**Missérété**	288	78	27,08	5	4	80	< 0,05
**Sèmè**	388	138	35,57	0	28	75,68	< 0,05
**Control**	88	43	48,86	0	65	36,52	0,05

* Total No * Total number; * Nber exit window trap * Number exit window trap.

### Variation in induced exophily in *Mansonia spp* and *Culex spp* in localities under IRS coverage

Exophily rate observed in *Mansonia spp* in Adjohoun (59.20%) and Sèmè (51.43%) was significantly different from that observed before intervention (p <0.05). In Dangbo district, there was no significant difference between these two rates (p = 0.196) (Table 5).

**Table 5:** **Exit index on* Mansonia spp and Culex spp *by bendiocarb treated walls before and after IRS intervention**

**Localities**	*** Mansonia spp. ***							*** Culex spp. ***						
	**Before intervention (May-July 2008)**			**After intervention (May-July 2010)**			**p-value**	**Before intervention (May-July 2008)**			**After intervention (May-July 2010)**			**p-value**
	**Total No**	**Nber exit window trap**	**Exit rate%**	**Total No**	**Nber exit window trap**	**Exit rate%**		**Total No**	**Nber exit window trap**	**Exit rate%**	**Total No**	**Nber exit window trap**	**Exit rate%**	
**Adjohoun**	128	20	15,63	27	16	59,26	p < 0,05	132	20	15,15	67	46	68,66	< 0,05
**Dangbo**	20	8	40	139	77	55,4	p = 0,196	56	20	35,71	198	67	33,84	0,79
**Missérété**		-	-	37	29	78,38	-	-	-	-	11	2	18,18	-
**Sèmè**	92	12	13,04	70	36	51,43	p < 0,05	177	49	27,68	83	26	31,33	0,54
**Control**				228	37	16,23					52	33	63,46	

* Total No * Total number.

 In *Culex spp *, it was only in Adjohoun that a significant difference was observed between the exophily rates before and after intervention (p <0.05). On the other hand, in Dangbo and Sèmè the exophily rate after IRS was not significantly different from before intervention (p = 0.79 and p = 0.54 respectively) (Table 5).

### Variation in mosquito blood feeding rate in *An. gambiae* in localities under IRS coverage

The blood feeding rate observed after IRS in the treated areas was low (Table 6). Compared to that observed before intervention, the difference between the two rates is significant (p <0.05) in all districts (Table 6). This rate was 9.09% after IRS against 54.76% before in Dangbo, 20% against 62.5% in Missérété and 35.14% against 54.90% in Sèmè.

**Table 6:** **Percentage of blood feeding* An. gambiae *collected in IRS by Pyrethrum Spray Catch (PSC) and in exit windows traps before (May-July 2008) and after (May-July 2010) interventions**

**Localities**	**Before intervention (May-July 2008)**			**After intervention (May-July 2010)**			**p-value**
	**Total No**	**Feed**	**Blood feeding rate (%)**	**Total No**	**Feed**	**Blood feeding rate (%)**	
**Adjohoun**	84	44	52,38	0	0	-	-
**Dangbo**	84	46	54,76	22	2	9,09	< 0,05
**Missérété**	288	180	62,5	5	1	20	< 0,05
**Sèmè**	388	213	54,9	37	13	35,14	< 0,05
**Control**	88	52	59,09	178	112	62,92	> 0,05

* Total No * Total number, * IRS * Indoor Residual Spraying.

## Discussion

The strategy of indoor residual spraying (IRS) implemented by the National Malaria Control Program (NMCP) in the Ouémé department with the PMI support had a great impact on malaria transmission. In all districts under intervention, the density of *An. gambiae* (human biting rate) and the entomological inoculation rate (EIR) have significantly declined.

The results have shown that *An. gambiae* s.s. M form was the major malaria vector species biting in the study area. The same trend was found by Padonou *et al.*[23], which explained the absence of the S molecular form by the ecological characteristics of the Ouémé region.

After the implementation of the third round of IRS, the biting rate of anopheles dropped drastically. In Adjohoun, Dangbo and Missérété, the mosquito biting rates were less than 2 bites per person per night. This drastic drop is due to the lethal effect of bendiocarb on the anophelines resistant to pyrethroids [16].

Indeed, comparing the number of *An. gambiae* bites that a person receives in one night from April to July 2008 and April-July 2010, the rates were significantly reduced. These results were consistent with those of Akogbéto *et al.*[14] in the same study area.

Mansonia and Culex mosquitoes are abundant in the area. Indeed, the main objective is to determine the impact of indoor residual spraying on malaria transmission and on the behavior of anopheline mosquitoes, especially *An. gambiae*. But owing to the high proliferation of other mosquitoes in the study area especially *Mansonia spp*. and *Culex spp*., it was an opportunity to determine the impact of the IRS on the behavior of those mosquitoes. Missing this opportunity to check a possible change in Culicinae against the IRS would be a big loss of information that we could obtain with no supplementary effort.

In the area, *Culex quinquefasciatus* is highly resistant to pyrethroids. In spite of this resistance, the IRS significantly reduced the density of these species of mosquito or increased exophily. These results indicated a successful prospect concerning the use of bendiocarb. Certainly, as shown in the article, many of the *Mansonia spp* and *Culex spp* bite more outside than inside and enter houses and take their blood meal on man in the bedroom before going out. Therefore, the increase of *Mansonia spp* exophily in the IRS area could be comparative with the control area. But the impact of the third round of IRS is very low.

In *Mansonia spp*, a lower human biting rate was observed in all districts after the introduction of the third round of IRS. But in *Culex spp*, the reduction observed was very low. This could be due to multiple mechanisms of insecticide resistance present in the *Culex spp*[24]. Performance noted are also linked to higher coverage of the IRS (the coverage rate of the first round is 90%).

During the April-July 2008 period (before intervention), the entomological inoculation rate (EIR) was estimated at 64.8, 68.4 and 54 infected bites per year for *An.gambiae* respectively in Adjohoun, Dangbo and Missérété. These data were similar to those observed in similar areas in southern Benin in 1990 [25,26] and in the city of Bouake in Cote d'Ivoire (67.3 infective bites per year) [27]. However, they were low compared to data recorded in Bancoumana, Mali (245 infective bites per person between June and October) [28]. Despite the relatively low EIR in the study area, in Sèmè and the control area (Adjara), the values were high (Table 1). These high rates of EIR were justified by the lack of LLINs in these environments.

The EIR was low in localities under IRS coverage. This shows that the IRS performed in four districts were a great success. Indeed, we had a reduction of 74.26% of EIR in the third round of IRS. However, in the work of Akogbéto *et al.*[14], this reduction was more than 94%. The efficacy of bendiocarb for large scale IRS was recently also demonstrated in Equatorial Guinea for *An. gambiae* where no infective mosquitoes were identified [29]. The difference between the reduction rates observed could be explained by the time between the second round (March 2009) and the third round (April 2010) of IRS. Indeed, in view of the low persistence of bendiocarb on the walls (3 to 4 months), after one year of IRS cessation, the people received infected bites of *An. gambiae* before the third round of PID. It is normal that after 12 months of IRS cessation, transmission resumes. This explains why 40% of mosquitoes positive for ELISA CSP were collected in April 2010 early in third round of IRS.

The EIR reduction is not only due to the large area covered by the IRS and the large protected population (500,000 inhabitants), the lethal effect of bendiocarb on mosquitoes resistant to pyrethroids [6,16], but also to the use of ITNs by children under five and pregnant women. The IRS area has become unfavourable for anopheles that died probably before the completion of the sporogonic cycle of *Plasmodium* due to a shorter longevity of *An. gambiae* in intervention areas. The effect of the carbamate bendiocarb on the walls inside a structure is harmful to mosquitoes. This could explain the high induction of exophily observed in *An. gambiae* and *Mansonia spp* (Tables 4 and 5). This high exophily induced by bendiocarb prevents mosquitoes from eating normally. This statement recalls the work of experimental huts of Asidi *et al.*[30], Darriet *et al.*[31] and N'Guessan *et al.*[32]. Indeed, some anopheles who managed to enter the houses could not take their blood meal before going out, which explains the very low blood feeding rate observed during the study (Table 6). However, very few mosquitoes are able to take their blood meal on the hosts in the house. The implementation of IRS should not exclude the use of LLINs by children and pregnant women.

## Conclusion

The three IRS campaigns in the Ouémé department with the PMI support had a significant impact on malaria transmission in the treated areas. The results show that in the four districts, the human biting rate of mosquitoes dropped spectacularly. A reduction of 74.26% in the entomological inoculation rate was recorded after three rounds of IRS, due to the decline in the lifespan of *An. gambiae* in treated areas and high exophily induced by bendiocarb on mosquitoes. However, a combined use of the IRS and LLINs will further reduce the contact between humans and mosquitoes and therefore, the total decrease of transmission and the blood feeding rate. Such a strategy implicates an increase in the cost of malaria prevention and cannot be implemented everywhere. It must be reserved only for areas with the highest malaria transmission.

Implementation of IRS in the department of Ouémé with the support of PMI is a success. This is why the National Malaria Control Program (NMCP) decided to continue to implement this strategy in the Atacora department and other regions of Benin after Ouémé experience.

## Competing interests

The authors declare that they have no competing interests.

## Authors’ contributions

RO, RA, GGP, AY and MA designed the study. RO and OO carried out the field activities. RO drafted the manuscript and analyzed the data. MA, RO and RA critically revised the manuscript. MA conceived and designed the study and revised the manuscript for intellectual content. All authors read and approved the final manuscript.
